# REM Sleep Impairment May Underlie Sleep-Driven Modulations of Tinnitus in Sleep Intermittent Tinnitus Subjects: A Controlled Study

**DOI:** 10.3390/ijerph20085509

**Published:** 2023-04-14

**Authors:** Robin Guillard, Louis Korczowski, Damien Léger, Marco Congedo, Alain Londero

**Affiliations:** 1 GIPSA-Lab, Grenoble INP, CNRS, Université Grenoble Alpes, 38000 Grenoble, France; 2 Robin Guillard EIRL, 38000 Grenoble, France; 3 VIFASOM ERC 7330, Vigilance Fatigue Sommeil et Santé Publique, Université Paris Cité, 75004 Paris, France; 4 Centre du Sommeil et de la Vigilance, Hôtel-Dieu, APHP, 75004 Paris, France; 5 Service ORL et Chirurgie Cervico-Faciale, Hôpital Européen Georges-Pompidou, APHP, 75015 Paris, France

**Keywords:** sleep intermittent tinnitus, sleep, REM, polysomnography, sleep apnea

## Abstract

(1) Background: Poor sleep and fragmented sleep are associated with several chronic conditions. Tinnitus is an auditory symptom that often negatively combines with poor sleep and has been associated with sleep impairment and sleep apnea. The relationship between tinnitus psychoacoustic characteristics and sleep is still poorly explored, notably for a particular subgroup of patients, for whom the perceived loudness of their tinnitus is highly modulated by sleep. (2) Methods: For this observational prospective study, 30 subjects with tinnitus were recruited, including 15 “sleep intermittent tinnitus” subjects, who had reported significant modulations of tinnitus loudness related to night sleep and naps, and a control group of 15 subjects displaying constant non-sleep-modulated tinnitus. The control group had matching age, gender, self-reported hearing loss grade and tinnitus impact on quality of life with the study group. All patients underwent a polysomnography (PSG) assessment for one complete night and then were asked to fill in a case report form, as well as a report of tinnitus loudness before and after the PSG. (3) Results: “Sleep Intermittent tinnitus” subjects had less Stage 3 sleep (*p* < 0.01), less Rapid-Eye Movement (REM) Sleep (*p* < 0.05) and more Stage 2 sleep (*p* < 0.05) in proportion and duration than subjects from the control group. In addition, in the “sleep Intermittent tinnitus” sample, a correlation was found between REM sleep duration and tinnitus overnight modulation (*p* < 0.05), as well as tinnitus impact on quality of life (*p* < 0.05). These correlations were not present in the control group. (4) Conclusions: This study suggests that among the tinnitus population, patients displaying sleep-modulated tinnitus have deteriorated sleep quality. Furthermore, REM sleep characteristics may play a role in overnight tinnitus modulation. Potential pathophysiological explanations accounting for this observation are hypothesized and discussed.

## 1. Introduction

Poor sleep has been associated with the onset or worsening of several chronic conditions [1,2,3,4]. Notably, insufficient sleep and poor sleep quality often seem to interact with the chronic condition creating an aggravating vicious circle, as described, for instance, in the case of type 2 diabetes [5], obesity and cardiovascular diseases [6]. Such a mutually reinforcing negative dynamic has also been described for poor sleep quality and tinnitus [7,8,9,10]. Tinnitus is defined as “the conscious awareness of a tonal or composite noise for which there is no identifiable corresponding external acoustic source” [11]. This chronic condition is highly prevalent, affecting approximately 14% of the world population, 2% of which are affected in an intrusive and debilitating fashion [12].

Regarding sleep and tinnitus, several authors have shown an association between subjective insomnia and tinnitus [13,14,15,16]. In a specific review [17], Asnis et al. highlight that although insomnia is strongly co-prevalent with tinnitus, too few studies give details on which sleep characteristics (difficulty falling asleep, awakenings during the night and early morning awakening) are more typically associated with it [14,18,19,20,21,22,23]. A couple of these studies reported that all aspects of insomnia were present in tinnitus patients, most severely, the difficulty in falling asleep [18,19].

Several studies have also objectively analyzed the sleep of tinnitus patients through polysomnography (PSG) [24,25,26,27,28,29,30]. These studies converged on the fact that objective sleep is impacted in tinnitus patients [26]. Two of these studies found that Rapid-Eye Movement (REM) sleep in tinnitus patients was significantly reduced [25,27]. One study was performed on a selected tinnitus patient population that concomitantly suffered from Post-Traumatic Stress Disorder (PTSD). It concluded that REM sleep proportion and duration were inversely correlated with Tinnitus Functional Index (TFI) scores [30]. Another found that the electroencephalography (EEG) delta-band power of tinnitus patients was reduced compared to that of the control group [24]. A fifth found that sleep efficiency and total sleep time were significantly reduced for tinnitus patients, and the number of awakenings significantly increased [29]. In order to explain this multiform phenomenon, two publications theorized that sleep impairment and tinnitus could share a common mechanism based on the hyperarousal theory [10,31].

Additionally, several studies have tried to evaluate the co-prevalence of tinnitus with known sleep disorders, such as bruxism [32,33], sleep apnea [34,35], periodic limb movements during sleep [36], snoring [28] and night terrors [37]. These studies highlighted the strong co-prevalence between tinnitus, bruxism and sleep apnea. 

A vast majority of these publications, whether focusing on sleep impairment or sleep disorders and tinnitus, analyzed these sleep characteristics in the tinnitus population without restrictively selecting a specifically homogeneous population, although it has been repeatedly suggested that tinnitus is a very heterogeneous symptom [38,39,40,41]. Keeping in mind that tinnitus is a highly heterogeneous condition, it is very unlikely that its interactions with sleep are consistent in all subgroups of tinnitus patients, as it is unlikely that a one-size-fits-all solution would be expected for such a highly heterogeneous symptom [42,43,44]. This is why studies aiming at understanding how sleep is impacted for some specific homogeneous tinnitus subpopulations may yield more decisive results.

Within the tinnitus population, there are two overlapping groups of patients for whom sleep plays a key role in their symptomatology. The first group describes significative and durable elevations of the tinnitus perceived loudness when awakening from naps [45,46]. This fact has been quite noticeable in clinical practice, as demonstrated by the fact that multiple studies have included this feature in their questionnaires [47,48,49]. A second, less numerous group of patients has been described in the scientific literature as “intermittent tinnitus” [50,51]; these patients display very significative modulations of tinnitus loudness after a night’s sleep. In this paper, this former group is referred to as sleep Intermittent tinnitus (SIT). They are specifically differentiated from patients exhibiting intermittent tinnitus, where the onset and offset of tinnitus are not linked to sleep occurrences. Such patients report that each day, upon awakening, the loudness of their tinnitus is assigned to a high or a low level, in a pseudo-cyclic pattern of 2 to 7 days or even a month in rare cases, and that this level of tinnitus loudness remains stable until the following sleep occurrence (night or nap), after which it may change again. In some cases, these patients report the complete disappearance of their tinnitus on certain days. This tinnitus subgroup also reports that if they have a nap on days when they have a low (or absent) tinnitus, there is a high probability that their tinnitus activates or increases in loudness upon awakening. Sleep fragmentation is also a common complaint in this subgroup of patients, which complains of frequent awakenings caused by their tinnitus onset.

In this second subgroup of SIT patients, sleep and its alteration seem to play a significant role in the modulation of tinnitus loudness, yet it has been poorly described and even less explored quantitatively. Therefore, the aim of our study is to assess the PSG sleep characteristics of this SIT population, compare them with subjects having stable tinnitus and explore links between PSG characteristics and tinnitus sleep-induced modulations.

## 2. Materials and Methods

### 2.1. Participants

Two cohorts of, respectively, 15 SIT patients (mean ± standard deviation age: 50.67 ± 10.83, 10 men, 5 women) and 15 stable, permanent chronic tinnitus forming the control group of patients (48.67 ± 13.26, 9 men, 6 women) and referred hereafter as Non-SIT group, were recruited to participate for a one-night polysomnography examination at Hôtel-Dieu hospital sleep department (VIFASOM) in Paris, France. Most of these patients were recruited by ENT doctor A. L. Some spontaneously enrolled in the study after a communication was made on social media platforms with the help of both the French patient tinnitus association France-Acouphènes and the mutual help digital community Siopi [52].

In both cohorts, patients had chronic tinnitus (over 6 months duration) and had either no tinnitus treatment or had a stable treatment for at least 3 months. Patients with Menière disease, acoustic schwannoma or pulsatile tinnitus were excluded from the study, as well as patients who reported epilepsy seizures and non-stabilized metabolic or cardiovascular conditions.

The SIT patients were all selected after thorough anamnesis with the investigators, the distinctive core trait of their tinnitus being that they all described strong modulations in relation to their sleep. Conversely, Non-SIT patients displayed stable non-fluctuating tinnitus without any modulation induced by night sleep or naps. 

To the best of our ability, gender, age, and tinnitus intrusiveness measured by the THI and self-declared hearing loss grade were matched between groups. The etiological characteristics of each arm are presented in Table 1.

All patients gave their informed consent to participate in the present clinical trial, which received approval from the ethical committee on insomnia and artificial intelligence (CPP Idf2 2018 A01034-51).

One subject manifested the desire to go deeper in the exploration of his sleep and volunteered to perform six additional nights of polysomnographic measurements. The longitudinal exploration of this subject’s sleep and associated tinnitus modulation is presented as a case report in Appendix A.

### 2.2. Clinical Assessment

#### 2.2.1. Anamnesis Case Report Form

The participants of both arms completed an exhaustive Case Report Form (CRF). This CRF included questions about tinnitus modulations over time, its somatosensory modulations, history of comorbidities and associated pain symptoms (jaw, cervical area and headaches). It also contained screening questions on sleep disorders and how tinnitus was modulated according to night sleep and naps. Lastly, this CRF also included the Tinnitus Handicap Inventory (THI). The THI is a 25-item questionnaire yielding a score between 0 and 100 classically used in tinnitus clinical studies to assess the impact of tinnitus on quality of life [53]. The main clinical characteristics of both samples are presented in Table 1.

#### 2.2.2. Overnight Tinnitus Variation Assessment

All participants filled out two short questionnaires on the night of their polysomnographic recording; one before going to bed and a second one shortly after awakening. These two questionnaires assessed tinnitus loudness and its associated intrusiveness using a visual analog scale (respectively, VAS-L and VAS-I), as well as the measures of the minimum masking levels (MML) of their tinnitus. MML was assessed with an MP3 device (Portable HD audio player, HIFI WALKER HX) and intra-auricular AGPTEK sleep headphones. The masking stimulation was a broadband white noise (20 Hz to 10 kHz). MML procedure started without any stimulation, and then the procedure progressively raised the masking stimulation intensity to the level that tinnitus was masked. The precise direction given to the participants was: “stop at the volume where you cannot discriminate your tinnitus perception from the sound stimulus, without actively focusing to do so”. This procedure for MML assessment was performed and reported three consecutive times in order to ensure the stability of the measurement.

In addition to these questionnaires, the short morning survey included a sleep agenda. Patients were asked to estimate the time at which they fell asleep and later awoke, as well as the frequency and estimated time of any additional nocturnal events overnight. 

#### 2.2.3. Polysomnography Recording

Polysomnographic recordings were performed using two Nox A1 devices (Resmed, San Diego, CA, USA). The sampling rates of both devices were 200 Hz and 250 Hz. EEG channels were positioned as usual at scalp locations F3, F4, C3, C4, O1, O2, M1 and M2 according to the 10–20 system for electroencephalography (EEG) positioning [54]. Two EOG channels were positioned diagonally: one externally above one eye and the other externally under the other eye. As well as three EMG sensors, two placed on the right and left suprahyoid muscles and one on the chin as reference.

All EEG, EMG and EOG electrodes were gold cup electrodes filled with conductive paste (Weaver and Company—Ten20 Conductive Neurodiagnostic, Aurora, CO, USA). The skin was initially cleaned with alcoholic solution (Alcohol 70% vol.) and dried before applying the conductive paste and positioning the electrodes. Electrodes were held in place using adhesive conductive cream (Natus Genuine Grass EC2 electrode cream, Middleton, WI, USA) on the top and adhesive bands (Mölnlycke—Mefix—Self-adhesive fabric Gothenburg, Sweden).

Two ECG sensors were placed on left arm/right leg positions. Single-use humid electrodes were used as reference (on the forehead) and for ECG measurement (Medtronics—Kendall—hydrogel ECG electrodes H92SG, Watford, UK).

The Nox A1 units are equipped with additional sensors: accelerometers in the 3 dimensions, activity measurement, angular position of the device and dB audio measurement.

Sleep apnea was assessed by means of two plethysmography belts on the thorax and abdominal regions. Furthermore, oximetry and pulse were measured continuously by NONIN 3150 Bluetooth oximeter (Nonin: Plymouth, MN, USA) and nasal airflow was measured by a nasal cannula connected to the pressure sensor of the polysomnographic NOX A1 device.

### 2.3. Analyses

#### 2.3.1. Polysomnography Scoring

Polysomnographic recordings were scored for sleep stages and sleep apnea following the ASSM recommendations by two experienced PSG technologists. For bruxism scoring, automatic scoring was first performed with the Tinnitus-n-sleep toolbox (https://github.com/lkorczowski/Tinnitus-n-Sleep accessed on 20 October 2022) and then reviewed manually. This toolbox automatically labels EMG jaw muscle signals for EMG bursts and scores bruxism events accordingly, following the recommendations given by Lavigne et al. [55,56]. This task was performed while rejecting artifacts provoked by body movements (measured by the accelerometer embedded on the NOX A1) and faulty electrode contact with the skin (measured by electrode impedance). It is important to acknowledge that, due to the COVID-19 pandemic, the use of a nasal cannula was not always possible. During this event, sleep apnea was not assessed, resulting in a restricted sample size for the analysis of this syndrome.

#### 2.3.2. Statistical Analyses

Data were centralized on a common anonymized CSV (Comma Separated Value) file and analyzed using Python and Scipy library [57]. The Mann–Whitney U test and Cohen D effect size were used to compare samples between groups. Spearman correlation tests were performed for each group between these sleep characteristics that exhibited significant differences between groups and overnight variations of tinnitus measured by MML, VAS-L and VAS-I scales.

## 3. Results

### 3.1. Etiological Group Comparison

Although the groups were matched by age, gender, self-declared hearing loss grade and tinnitus impact on their quality of life, as measured by the THI, differences were identified between groups on several CRF items, as presented in Table 1. The identified differences between groups were as follows:

- The proportion of patients experiencing an increase of tinnitus after naps were significantly higher in the SIT group than in the Non-SIT group (*p* < 0.01);
- The proportion of patients complaining of neck stiffness and/or lack of cervical mobility was significantly lower for the SIT group than for the Non-SIT group (*p* < 0.01);
- The proportion of patients experiencing rises of tinnitus after an exposition to loud noises was significantly lower in the SIT than in the Non-SIT group (*p* < 0.05).

**Figure 1:**
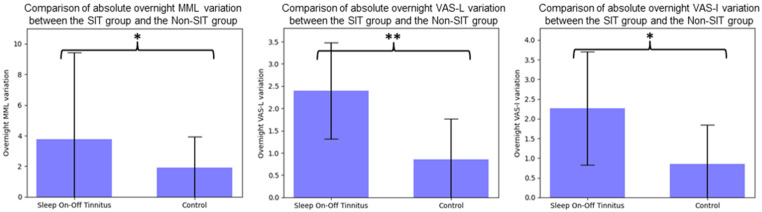
Comparison of absolute overnight tinnitus variation between groups, MML: Minimum Masking Level, VAS-L: Visual analog scale on tinnitus loudness, VAS-I: Visual analog scale on tinnitus intrusiveness. *: *p* < 0.05, **: *p* < 0.01.

Moreover, according to our expectations, overnight absolute variations of tinnitus level, measured by MML and VAS-L and VAS-I scales, were all significantly higher in the SIT group compared to the matched sleep-stable tinnitus Non-SIT group (*p* < 0.05 for MML and VAS-I, *p* < 0.01 for VAS-L), as highlighted in Figure 1.

During clinical interactions with SIT patients, at least one-third of them spontaneously reported a noteworthy phenomenon: whenever they woke up early in the morning with low or absent tinnitus, if by chance they went back to sleep even for a short period of time after this first awakening, then their tinnitus would almost systematically increase upon their second awakening. None of the patients in the Non-SIT group reported such a phenomenon.

### 3.2. Sleep Characteristics Group Comparison

Among the Non-SIT group, one subject underwent the complete one-night polysomnography procedure yet slept less than three hours. Consequently, this subject’s recording was excluded from the analysis. Due to the COVID-19 pandemic’s sanitary prerogatives, airflow cannulas could not be used for five recordings (two of the SIT group and three of the Non-SIT group). Consequently, regarding the group comparison over the sleep apnea criterion, these recordings were excluded from analyses.

Polysomnographic sleep characteristics of the SIT group and the Non-SIT group are presented in Table 2. Significant differences in percentages of total sleep time of N2 stage (SIT: 53.87 ± 10.79, Non-SIT: 44.74 ± 6.5, *p* < 0.05, Cohen D: 0.98), N3 stage (SIT: 22.93 ± 10.97, Non-SIT: 28.52 ± 5.88, *p* < 0.01, Cohen D: 0.61) and REM stage (SIT: 15.93 ± 5.84, Non-SIT: 21.08 ± 4.66, *p* < 0.05, Cohen D: 0.94) were identified between the SIT group and the Non-SIT group. Similarly, significant differences between sleep duration (in min) of the N3 stage (SIT: 86.45 ± 35.18, Non-SIT: 114.89 ± 22.8, *p* < 0.01, Cohen D: 0.92) and REM stage (SIT: 62.13 ± 25.46, Non-SIT: 87.18 ± 26.5, *p* < 0.05, Cohen D: 0.93) were identified between the SIT group and the Non-SIT group.

**Figure 2:**
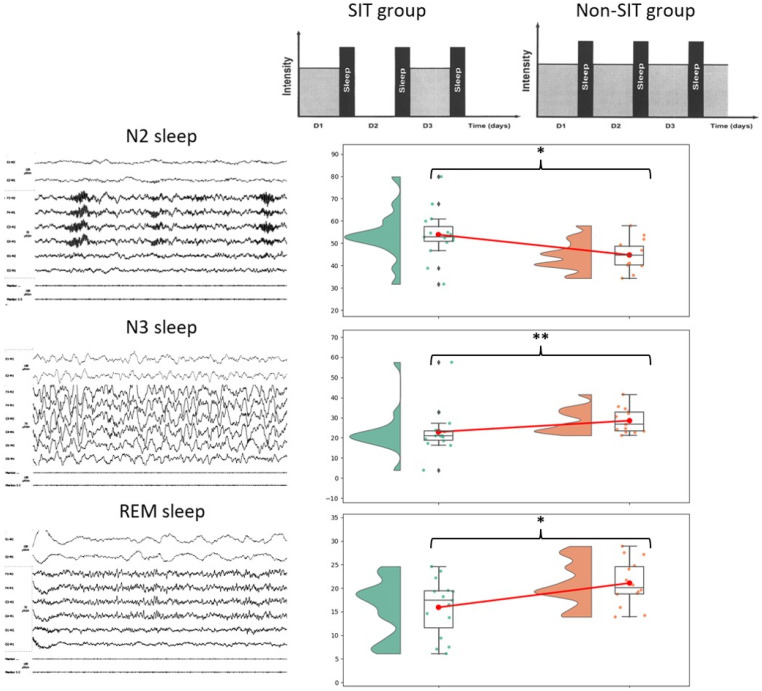
General overview of sleep characteristics main difference between SIT group and matched Non-SIT group. Tinnitus dynamic display figure adapted with permission from Ref. [50]. 2002, Cecile Nicolas-Puel. *: *p* < 0.05, **: *p* < 0.01.

An overall view of the group differences is displayed graphically in Figure 2.

### 3.3. Correlations between Tinnitus Modulation and Sleep Characteristics

**Figure 3:**
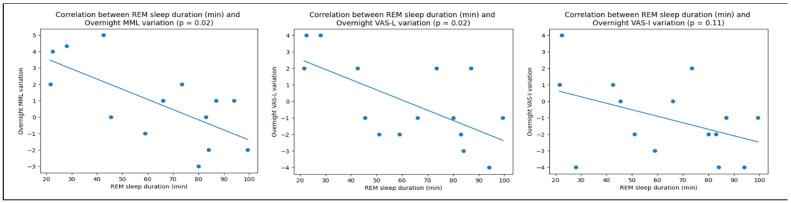
Anticorrelations between REM sleep duration (in min) and overnight tinnitus variations measured by Minimum Masking Level (MML), Visual Analog Scales on Tinnitus Loudness (VAS-L) and Intrusiveness (VAS-I).

Correlation tests were performed between N2, N3 and REM sleep stages percentages of total sleep time and duration (in min) and overnight tinnitus variation measured by MML, VAS-L and VAS-I. Significative correlations were found only in the SIT group between REM sleep duration and overnight MML (*p* < 0.05) and VAS-L (*p* < 0.05) variation. These correlations are displayed in Figure 3. A similar significant correlation was found between REM stage percentage of total sleep time and overnight VAS-L variation (*p* < 0.05) for the longitudinal case report presented as Appendix A. A significant correlation was also found between the THI scores and the REM sleep duration (in min) in the SIT group (*p* < 0.05).

## 4. Discussion

This study aimed to understand the sleep characteristics of a specific subgroup of tinnitus patients presenting important sleep-driven modulations of their tinnitus percept, referred to as SIT patients. It has been repeatedly reported that tinnitus patients are often sleep deprived and/or suffer from impaired quality of sleep [13,14,15,17,29]. These studies have analyzed heterogeneous samples of the tinnitus population, neglecting de facto its reported intrinsic heterogeneity [44,58,59]. To the best of the authors’ knowledge, the present study is the first to analyze the interactions between sleep and tinnitus in two homogeneous subgroups of tinnitus patients matched in gender, age, tinnitus intrusiveness and self-declared hearing loss grade.

In the present study, 15 SIT patients and 15 matched sleep-stable tinnitus Non-SIT patients participated in a one-night polysomnography assessment. Comparative analyses of the sleep characteristics of each group confirmed specificities in the sleep characteristics of SIT patients. The latter exhibited deteriorated sleep compared to Non-SIT patients as they presented more N2 stage (light sleep), less N3 stage (deep sleep) and less REM stage sleep. On the other hand, no significant differences were found between groups for sleep latency or wake time after sleep onset. This tends to highlight that the differences between groups do not seem linked to an underlying difference in sleep quantity but instead to sleep quality. Moreover, as no significative differences were identified between groups for sleep apnea (measured by Apnea-Hypopnea Index and Oxygen Desaturation Index), this comorbidity does not seem to account for the SIT specificities. Additionally, it is important to mention that, although not significant, a difference between groups on sleep bruxism was noted with a medium Cohen-D effect size. However, this difference highlighted that SIT patients actually had fewer sleep bruxism episodes per hour as compared to Non-SIT patients. One possible explanation could be that SIT patients experience more intense temporo-mandibular joint (TMJ) and/or facial pain than Non-SIT patients. In fact, in their study, Rompré et al. showed that such pain is associated with lower frequencies of orofacial activities [60]. In the present study, this hypothesis seems to be strengthened by the fact that etiological items group comparison shows that SIT group experienced slightly more facial and/or TMJ pain compared to the Non-SIT group (yet not significative) and more TMJ muscle fatigue (*p* = 0.08, medium effect size of 0.63). Future research at a larger scale should test this hypothesis further.

### 4.1. REM Sleep Seems to Be Specifically Implicated in SIT Sleep-Driven Modulations

Before starting this prospective study, A.L. and R.G. collected polysomnography reports over the years of several SIT patients who spontaneously performed such assessments to try to understand why their tinnitus was influenced by their sleep. These preliminary aggregations of sleep reports (not published) pointed to an impairment of REM sleep.

The present study confirmed that this specific tinnitus subgroup had less REM sleep proportion and duration (*p* < 0.05) than matched Non-SIT patients. It is important to mention that, according to Ohayon et al. [61], for a healthy sample of the population of mean age 50 years old, the proportion of REM sleep over total sleep time should be around 20%. Yet, the SIT sample of the present study had 15.9% of REM sleep, which appears sensibly lower. In comparison, the Non-SIT group averaged a normal 21% of REM sleep.

It is also important to mention that two former publications on polysomnographic analyses on tinnitus patients [25,27] found congruent findings that REM sleep appeared quantitatively deteriorated in the tinnitus population compared to healthy Non-SIT patients, although these authors did not specifically select SIT patients nor reported their representative proportion in their samples. 

Another interesting finding is the correlation observed between the duration of REM sleep during the polysomnographic assessment and the variation of tinnitus overnight, measured by MML and VAS-L in the SIT group. Likewise, the same significative correlation was found across seven nights on the same subject in the longitudinal case report presented in Appendix A. It is interesting to note that such a correlation was found for measurements accounting for psychoacoustics characteristics of the tinnitus percept only and not for the VAS-I, which accounts for intrusiveness and hence the psychological reaction to the percept. 

However, the additional fact that a significative correlation was found between THI scores of SIT patients and their REM sleep duration is puzzling and harder to interpret. In fact, by its definition, people affected by SIT have very different experiences depending on the days (both perceptually and emotionally), and yet the THI asks general questions that address tinnitus “on the average of time”. Hence, this questionnaire is probably not fit to account for the tinnitus inter-day variability of this specific tinnitus subgroup. One could hypothesize that SIT patients answer the THI questionnaire picturing themselves on days when their tinnitus is present and bothers them. If we take this hypothesis for granted, the observed correlation could then suggest that the more REM sleep deteriorates in SIT patients, the more tinnitus impacts the quality of life of these patients on loud tinnitus days.

The observed correlation between THI scores and REM duration in the SIT group is quite similar to the recently reported significant correlation between Tinnitus Functional Index (TFI) scores and REM sleep duration and proportional in-total sleep time in Post-Traumatic Stress Disorder (PTSD) patients experiencing tinnitus [30]. This correlation is all the more interesting, considering the parallels that can be drawn between PTSD and tinnitus. The co-prevalence and association between tinnitus and PTSD are notorious [30,62,63,64]. Additionally, it is noteworthy that most tinnitus patients maintain a precise memory of the moment when they first experienced their tinnitus, which could be thus assimilated to a traumatic and stressful event. This could explain why treatments such as Eye Movement Desensitization and Reprocessing (EMDR) therapy have repeatedly been suggested as a potentially good approach to help tinnitus patients [65,66]. Two reviews on EMDR can be found here: [67,68]. Although the PTSD relation to REM sleep’s polysomnographic characterization yields conflicting results, recent findings in human sleep and, above all, rodent sleep identified that REM sleep could somehow be maladaptive in PTSD cases, with abnormal levels of noradrenaline during sleep and early after awakening, resulting in abnormally elevated locus coeruleus firing during REM sleep [69]. It is currently postulated that locus coeruleus firing during REM sleep impedes the loosening of the emotional schema associated with the traumatic memory [70]. Whether or not SIT patients have similar pathophysiological responses as PTSD patients remains an open question for future research. Indeed, the CRF in the present study has not explored the psychological characteristics of the sample. It could also be interesting to test EMDR procedures on this specific population to see if it proves to be specifically effective.

The correlation between the impairment of REM sleep and SIT modulation poses the question of any causal relationship. 

Indeed, some studies have suggested that REM sleep could be impaired for healthy individuals exposed to continuous noise during their sleep [71,72,73]. In the SIT population, the onset of tinnitus during the night could damage REM sleep similarly to such continuous noise. 

Conversely, the present results could suggest that REM sleep impairment could cause the modulation of tinnitus. Such a hypothesis is undermined by two other observations. In fact, the SIT group reports significantly more frequent tinnitus nap modulations than the Non-SIT group. Still, REM sleep is uncommon during naps, except during morning naps [74,75] or at the end of long afternoon naps of more than 45 min duration [76]. However, SIT patients report rises in their tinnitus even after *short*-duration naps and *anytime* in the day. Another important point is that after night, SIT patients displayed important *rises or decreases* in their tinnitus, whereas after naps or after morning sleep prolongations, they only reported *rises*. Somehow, there seems to be a dissymmetry in the way sleep interacts with tinnitus during nights and during naps, and this suggests a potential circadian role. Such clinical observations from aggregated case reports need to be confirmed with rigorous prospective measurements. Yet, if proven right, it could thus be deduced that tinnitus sleep intermittent modulations can happen without the occurrence of REM sleep during naps.

Additionally, an underlying root pathophysiological mechanism could be the origin of both the modulation of tinnitus and the impairment of REM sleep, explaining this correlation. Such a phenomenon would also occur during naps and provoke, in these cases, only rises of the tinnitus percept. Such putative pathophysiological mechanisms could be facial muscle atonia modulations (neck and or jaw potentially linked to sleep position), sleep apnea or sleep bruxism and also, more interestingly, REM sleep middle-ear muscle activity (MEMA) of the Tensor tympani and/or Tensor Veli Palatini reported by [77,78,79,80].

### 4.2. Merits

To the best of the authors’ knowledge, the present study has the merit of being the first to take into account tinnitus heterogeneity for the exploration of polysomnographic characteristics of the tinnitus population. It is also the first to have objectivized, through polysomnography assessments, the sleep characteristics of tinnitus patients reporting important sleep-driven modulation of their tinnitus, referred to here as SIT patients. Another merit compared to past studies of the domain is that the present study precisely reported the etiological characteristics of each recruited group while also presenting not only their polysomnographic characteristics but also their sleep apnea and bruxism assessments. 

### 4.3. Limits

The first limitation of this study is its small sample size. It proved difficult to recruit SIT patients in the time span of the study, above all, as the study was monocentric. It seems the target subgroup of tinnitus patients only represents a small minority of tinnitus patients. This first limitation induced another one: due to the small sample sizes in this study, statistical rigorousness and conservative corrections (as, for instance, Bonferroni correction) could not be applied to account for the multiple tests performed in the analyses. Hence, only limited credit can be given to the conclusion of the present study and its statistical methodology. Future research should definitely try to independently reproduce the present work in order to try to confirm its results. 

The fact that polysomnography was only assessed during one night without a preliminary habituation night, nor actigraphy nor sleep diaries was another limitation to this study, although the E-CRF provided some declarative first elements on sleep duration, insomnia and snoring status. As a potential consequence, one Non-SIT group subject slept less than three hours and his/her night had to be excluded. 

Moreover, SIT patients describe diverging variations of their tinnitus across nights, only one of them being sampled in this study. Although an important variance of overnight tinnitus percept variation was captured in this study, it appears valuable in such circumstances to perform longitudinal measurements of each patient. The longitudinal measurement of one patient, presented in the case report in Appendix A, is a first step in this direction. Future research should try to generalize these longitudinal observations to the totality of their cohorts. 

Another methodological limitation lies in the measurement of overnight tinnitus variations. In fact, by measuring only MML, VAS-L and VAS-I at only two-time points, once before and once after the polysomnographic recording, one cannot know if the observed variation was important or insignificant according to the subject’s daily experience of tinnitus variations. It would bring additional value to track each subject the variation of their tinnitus percept over several days to have insight into their individual tinnitus variance and dynamic. Such individual longitudinal normalization would provide more validity to group correlations and effect size computations between objective physiological assessments and such overnight tinnitus variation measurements.

Likewise, in this study, groups were only matched on self-reported hearing loss grade. Such subjective criteria could be advantageously replaced in future research by objective audiological assessments such as at least an audiogram and perhaps more elaborated hearing tests. 

### 4.4. Suggestions for Future Research

In addition to previously mentioned suggestions for future research, it should be advised for future studies to define more objective selection criteria to discriminate SIT patients. Defining a threshold on the degree of variability of tinnitus loudness across several morning and evening measurements of MML or VAS-L appears to be a good approach to defining such a discriminative criterion.

Another suggestion in order to better understand the underlying pathophysiological mechanism provoking these intermittent sleep variations would be to switch from a night polysomnography paradigm to a short nap observation paradigm. In fact, as could be observed in the etiological analyses, the prevalence of nap-related modulations of tinnitus in the SIT population is striking. A protocol following a similar design yet organized for naps would enable us to narrow down more effectively the key mechanisms at play behind these modulations. Such short naps (15 min or less) would constitute a more focused approach to enable better control over adverse events and biases compared to 7 or 8 h night sleep recordings.

Similarly, following the latter discussion that a potential root pathophysiological mechanism modulating tinnitus affects both REM sleep and naps sleep, in such a nap condition new protocol, it could be interesting to multiply objective physiological and functional measurements before, during and after each nap. Notably, to complete previous suggestions, blood testing and functional imagery would be relevant additional measurements.

## 5. Conclusions

The present study presented the specific sleep characteristics of patients exhibiting important sleep-driven modulations of their tinnitus, referred to here as SIT patients, compared to matched Non-SIT patients. It showed that while there are no differences in sleep quantity nor in sleep apnea syndrome prevalences between the two groups, the SIT group’s sleep appeared more deteriorated than the sleep of the matched Non-SIT group. REM sleep deterioration potentially plays an important role in these modulations, as suggested by several correlations which were observed. Future research should further investigate these modulations on larger cohorts and in more longitudinal modalities to confirm these preliminary results.

## Figures and Tables

**Table 1:** Sample characteristics.

	SIT Group (*n* = 15)	Non-SIT Group (*n* =15)	*p*-Value	Effect Size
	Mean (Std)	Mean (Std)		
Gender ratio (W = 1; M = 0)	0.33 (0.47)	0.4 (0.49)	0.73	
Age (years)	50.67 (10.83)	48.67 (13.26)	0.71	
THI score (0 - 100)	54.4 (18.59)	47.87 (25.61)	0.38	
VAS score on tinnitus intensity (0, 10)	6.29 (1.67)	6.6 (1.54)	0.47	
VAS score on tinnitus annoyance (0, 10)	7.5 (1.64)	6.47 (2.31)	0.33	
Tinnitus pitch (High: 0, Medium: 0.5, Low: 1)	0.13 (0.34)	0.16 (0.35)	0.7	
Tinnitus lateralisation (Bilateral: 0, Partly lateral: 0.5, Unilateral: 1)	0.43 (0.32)	0.3 (0.4)	0.27	
Hearing loss grade (No: 0, Mild: 1, Medium: 2; Severe: 3)	0.73 (0.68)	0.87 (0.96)	0.95	
Have used or are using hearing aids (Yes: 1, No: 0)	0.47 (0.5)	0.53 (0.5)	0.74	
Tinnitus increases after naps (Strongly: 1, Mildly: 0.5, No: 0)	0.87 (0.29)	0.07 (0.17)	<0.01 **	3.499
Masked by noisy environment ? (Yes: 1, No: 0)	0.47 (0.5)	0.47 (0.5)	1.0	
Masked by white noise ? (Yes: 1, No: 0)	0.47 (0.5)	0.8 (0.4)	0.07	−0.712
Tinnitus raises with long noise exposure (Yes: 1, No: 0)	0.23 (0.36)	0.37 (0.43)	0.41	
Tinnitus raises with loud noise (Yes: 1, No: 0)	0.1 (0.2)	0.43 (0.44)	0.03 *	−0.938
Are daily noises inconfortable ? (Yes: 1, No: 0)	0.53 (0.5)	0.6 (0.49)	0.74	
Is powerful noise exposure painful ? (Yes: 1, No: 0)	0.47 (0.5)	0.6 (0.49)	0.49	
Jaw stiffness in the morning (Frequency gradation)	0.47 (0.81)	0.53 (0.88)	0.94	
Jaw clenching during the day (Frequency gradation)	1.0 (1.32)	0.4 (0.8)	0.2	0.532
Facial pain or jaw pain (Frequency gradation)	0.73 (1.0)	0.4 (0.8)	0.29	
Facial or jaw muscle fatigue (Frequency gradation)	0.6 (0.88)	0.13 (0.5)	0.08	0.631
Jaw popping (Frequency gradation)	0.67 (1.14)	0.67 (1.01)	0.88	
Headaches/migraine (Frequency gradation)	0.6 (0.95)	1.0 (1.15)	0.37	
Neck pain (Frequency gradation)	1.47 (1.54)	1.6 (1.08)	0.79	
Neck stiffness/hypomobility (Frequency gradation)	0.27 (0.77)	1.27 (1.24)	<0.01 **	−0.937
Ear fullness (Frequency gradation)	1.07 (1.06)	0.87 (1.2)	0.62	
Autophony (Frequency gradation)	0.13 (0.5)	0.4 (0.8)	0.31	
Otalgia (Frequency gradation)	0.6 (1.14)	0.73 (1.34)	0.94	
Sleep duration (<6 h: −1, 6–8 h: 0, >8 h: 1)	0.07 (0.57)	−0.07 (0.57)	0.55	
Self reported insomnia (Yes: 1, No: 0)	0.4 (0.49)	0.13 (0.34)	0.11	0.611
Hard to stay asleep (Yes: 1, No: 0)	0.6 (0.49)	0.6 (0.49)	1.0	
Hard to fall asleep (Yes: 1, No: 0)	0.27 (0.44)	0.47 (0.5)	0.27	
Snoring (Yes: 1, No: 0)	0.73 (0.44)	0.4 (0.49)	0.07	0.69

Std: standard deviation, Frequency gradation: (Always: 4, Often: 3, Sometimes: 2, Rarely: 1, Never: 0), *: *p* < 0.05, **: *p* < 0.01.

**Table 2:** Sleep characteristics comparison.

	SIT Group (*n* = 15)		Non-SIT Group (*n* = 14)		*p*-Value		Effect Size	
	Mean (Std)		Mean (Std)					
Total sleep time (TST, in min)	385.63 (46.37)		408.35 (61.11)		0.23			
Wake time after sleep onset (WASO, in min)	59.31 (35.32)		68.79 (38.72)		0.6			
Sleep latency (min)	16.13 (18.71)		18.45 (16.83)		0.33			
REM sleep latency (min)	120.87 (44.89)		118.68 (54.67)		0.66			
Total Bruxism (number of episodes per hour)	13.05 (4.14)		15.85 (5.29)		0.14		−0.571	
Phasic Bruxism (number of episodes per hour)	1.72 (0.69)		1.88 (0.85)		0.61			
Tonic Bruxism (number of episodes per hour)	3.02 (1.81)		4.53 (2.17)		0.06		−0.733	
Mixed Bruxism (number of episodes per hour)	0.77 (0.44)		0.93 (0.39)		0.31			
Apnea Hypopnea Index (AHI) (*)	14.19 (10.58)		14.0 (10.1)		0.91			
Oxygen Desaturation Index (ODI)	12.77 (10.16)		13.37 (8.71)		0.79			
	**% TST**	**Duration (min)**	**% TST**	**Duration (min)**	**For % TST**	**Duration (min)**	**For % TST**	**Duration (min)**
N1 sleep (%, min)	7.2 (2.79)	27.55 (9.71)	5.68 (2.74)	22.05 (7.85)	0.13	0.1	0.53	0.6
N2 sleep (%, min)	53.87 (10.79)	209.04 (53.31)	44.74 (6.5)	184.34 (44.87)	0.01 *	0.2	0.98	
N3 sleep (%, min)	22.93 (10.97)	86.45 (35.18)	28.52 (5.88)	114.89 (22.8)	<0.01 **	<0.01 **	−0.61	−0.92
REM sleep (%, min)	15.93 (5.84)	62.13 (25.46)	21.08 (4.66)	87.18 (26.5)	0.04 *	0.04 *	−0.94	−0.93
Snoring (%, min)	20.23 (15.45)	74.75 (54.26)	19.08 (19.19)	72.46 (72.29)	0.66	0.65		
Supine position (%, min)	45.33 (33.81)	185.18 (149.91)	30.86 (24.89)	127.4 (105.41)	0.25	0.37		
Lateral position (%, min)	48.47 (31.79)	178.9 (114.28)	56.29 (27.42)	223.7 (108.22)	0.53	0.31		
Prone position (%, min)	4.46 (8.82)	18.61 (36.32)	10.43 (18.07)	45.95 (81.85)	0.16	0.15		

Std: standard deviation, REM: Rapid Eye Movement, TST: total sleep time. (*): For AHI, SIT: *n* = 13, Non-SIT: *n* = 11. *: *p* < 0.05, **: *p* < 0.01.

**Figure A1:**
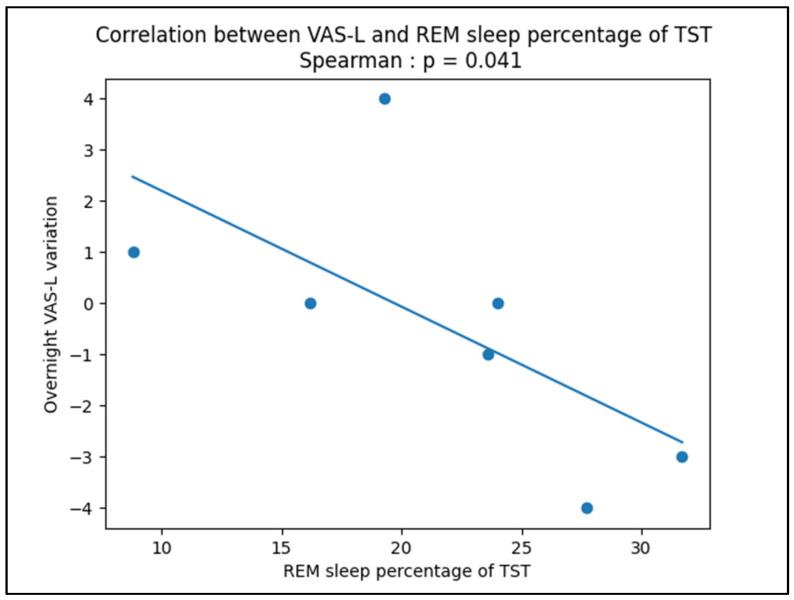
Anticorrelation between REM sleep percentage of total sleep time and overnight VAS-L variation, TST: total sleep time.

## Data Availability

Data available on request due to ethical restrictions.

## Appendix A

Longitudinal 7 days polysomnography assessment of a sleep intermittent tinnitus patient—a case report.

1. Subject Presentation

This appendix presents a case report of seven nights of longitudinal polysomnographic recordings of a 50-year-old 1.78 m man reporting sleep intermittent tinnitus (SIT). The subject had started presenting sleep intermittent modulations of tinnitus six months before his first polysomnographic participation. He presented a right unilateral high pitch (around 10 kHz) tinnitus that was continuous when present and yet that could totally disappear upon awakening and stay absent until a subsequent sleep occurrence. He described pseudo-cyclic though random alternances of 2 to 4 days “on” and “off” periods of tinnitus. The subject presented notable retrognathism without temporo-mandibular or headache complaint. According to his description, tinnitus could also sometimes rise spontaneously after lunch during the post-prandial period. He presented only slight somatosensory modulations of his tinnitus, as he described being able to reduce the tinnitus when advancing the jaw slightly. No other maneuver of the jaw or neck elicited any change in his tinnitus percept. The patient described important rises of his tinnitus after short naps, notably in the evening while his television remained on. He described his tinnitus as very bothersome in the first months after its onset and was prescribed 10 mg of mianserin per day to cope with his tinnitus. This treatment gradually helped him, yet without successfully suppressing tinnitus. When he applied to participate in the present study, his treatment was stable for more than three months, and his THI score was 36. The patient’s audiogram was normal for both ears, with a slight hearing loss of 8 kHz in both ears.

2. Polysomnographic Recordings and Methodology

The subject participated in the present clinical study and subsequently manifested the will to participate in further longitudinal explorations of his sleep in order to understand better why his tinnitus was such drastically influenced by sleep. He thus participated in two additional nights of polysomnography at the hospital in the same experimental conditions as the study. He was then lent one Nox A1 and all necessary materials to continue polysomnographic measurements at home. He was advised how to minimally install the material by himself to perform the polysomnographic recordings with autonomy. Consequently, for his recordings at home, C3, C4, O1 and O2 electrodes were not recorded; he did not use an airflow cannula for these recordings either. Five recordings were performed by the patient in autonomy with this setup.

Out of the eight recordings cumulated, seven were exploitable (a battery issue occurred in one of them) and were analyzed following the same methodology as in the observational group study. To score recordings when C3, C4, O1 and O2 were not available, the experimenters used bipolar channels F3-M2 and F4-M1 to assess sleep stages.

The subject did not record his MML levels during his polysomnographic recordings at home and hence only reported VAS-L and VAS-I measurements before and after each recording. In his opinion, VAS-L and VAS-I measurements were always identifiable and scored them always equally.

To try to evaluate the potential link between sleep characteristics and the overnight modulation of tinnitus, we performed spearman correlation tests.

3. Results

The sleep characteristics of the seven exploitable recordings are presented in Table A1. As can be seen, one night with a consequent rise of tinnitus was recorded (night 2), as well as two nights where tinnitus disappeared at the end (nights 4 and 5) and four nights where the loudness of tinnitus remained approximately the same. It could yet be pointed out that situations are maybe dissimilar between night 3, where tinnitus was absent before and after, and on night 6, where it was present with a loudness score of 3 on the VAS-L before and after the night.

**Table A1:** Longitudinal sleep characteristics.

	VAS-L Evening	VAS-L Morning	TST	WASO	Sleep Latency	REM Latency	%N1	%N2	%N3	%REM	%Snoring	%Supine
Night 1	2	1	421	8.3	5.3	71.5	4.9	38.8	32.7	23.6	10.7	97.4
Night 2	1	5	514.5	21.2	6.6	80	11	54.8	14.9	19.3	17.5	94.2
Night 3	0	0	423	22.6	7.7	70.7	6.4	39.4	38	16.2	6.1	78.9
Night 4	3	0	358	22.5	2.4	44.4	6	39	23.3	31.7	2.8	72.8
Night 5	4	0	464	27	8	70	5.6	37.4	29.3	27.7	3.6	75.9
Night 6	3	3	339	7.2	15.9	96.9	5.7	29.6	40.6	24	30.1	82.7
Night 7	0	1	297	2.9	2.4	91.4	3.5	32.3	55.4	8.8	30.6	75.6

Abbreviations: TST: total sleep time, WASO: wake time after sleep onset.

An important was observed variability in almost all sleep characteristics. While testing for correlations between sleep characteristics and overnight VAS-L variation, overnight VAS-L variation displayed a significative correlation with REM sleep percentage of total sleep time (anticorrelation, *p* < 0.05, illustrated in Figure A1) and with a percentage of snoring (correlation, *p* < 0.05).

4. Discussion

The present case report displays the longitudinal evolution of tinnitus across days in a patient identified to have SIT. As it could be observed, on 5 out of the 14 VAS-L measurement occasions, tinnitus was completely absent. On the other hand, when present, tinnitus loudness was scored by the subject between 1 and 5. The subject described that historically, his tinnitus could get louder than during the present recordings.

Polysomnographic characteristics of the patient revealed important (yet potentially normal) variability. Correlations were identified between the overnight tinnitus loudness variation measured by the VAS-L and the REM-sleep percentage of total sleep time. This result matches the similar anticorrelation observed over the whole group of SIT patients and is comforting the hypothesis that REM sleep may play a role in the observed tinnitus loudness variation. On the contrary, the observed correlation between the percentage of time snoring and overnight VAS-L variation has not been found for the group analysis.

Such longitudinal measurements appear all the more essential to carry out knowing the day-to-day variability of this specific type of tinnitus described by patients.
